# Prognostic predictive values of gemcitabine sensitivity-related gene products for unresectable or recurrent biliary tract cancer treated with gemcitabine alone

**DOI:** 10.1186/1477-7819-11-117

**Published:** 2013-05-27

**Authors:** Akihiro Murata, Ryosuke Amano, Nobuya Yamada, Kenjiro Kimura, Masakazu Yashiro, Bunzo Nakata, Kosei Hirakawa

**Affiliations:** 1 Department of Surgical Oncology, Osaka City University Graduate School of Medicine, 1-4-3 Asahi-machi, Abeno-ku, Osaka 545-8585, Japan

**Keywords:** Biliary tract cancer, Deoxycytidine kinase, Gemcitabine, Human equilibrative nucleoside transporter 1, Ribonucleotide reductase subunit M1

## Abstract

**Background:**

Gemcitabine is a pyrimidine nucleoside analog that is a commonly used chemotherapeutic agent for unresectable or recurrent biliary tract cancer (BTC). Several molecules involved in gemcitabine metabolism, including human equilibrative nucleoside transporter (hENT1), deoxycytidine kinase (dCK), and ribonucleotide reductase subunit M1 (RRM1), have been investigated as predictive biomarkers of gemcitabine efficacy, mostly in pancreatic cancer. The aim of this study is to clarify which biomarker is the most reliable among hENT1, dCK, and RRM1 to predict survival in patients with advanced BTC treated with gemcitabine alone.

**Methods:**

The analysis was performed on samples from 28 patients with unresectable or recurrent BTC who were treated with gemcitabine alone as first-line therapy. The starting date of overall survival (OS) and progression-free survival (PFS) was defined as the date of first treatment with gemcitabine. Intratumoral hENT1, dCK, and RRM1 expressions were examined by immunohistochemistry.

**Results:**

The expressions of hENT1, dCK, and RRM1 had no significant relationships with age, gender, primary tumor site, recurrence/unresectable, or histological type. Among the three molecules, only hENT1 expression was a significant factor affecting OS and PFS in univariate analysis; OS was 11.4 months for high hENT1 expression versus 5.7 months for low, *P* = 0.0057; PFS was 7.7 months for high versus 2.5 months for low, *P* = 0.0065. Multivariate analyses also identified hENT1 expression as an independent predictive factor for OS.

**Conclusions:**

hENT1 is the most reliable predictive marker of survival in patients with advanced BTC treated with gemcitabine.

## Background

Biliary tract cancer (BTC) is relatively rare, but its incidence is increasing worldwide. The prevalence is much higher in East Asia and Latin America than in Europe and the United States [1,2]. Although a complete surgical resection is the only curative modality, most patients are not eligible for surgery because of the advanced stage of disease at diagnosis. Moreover, even patients who undergo a surgical resection often have a recurrence of the disease. The outcome for patients with unresectable or recurrent BTC is dismal and their median survival is usually under 1 year [3]. Most patients with unresectable or recurrent BTC are therefore candidates for palliative chemotherapy.

Gemcitabine (2′,2′-difluorodeoxycytidine), a deoxycytidine analog that inhibits DNA replication and repair, is the most effective single agent in advanced BTC. Because gemcitabine is hydrophilic and does not cross the plasma membrane by passive diffusion, its cellular uptake requires the presence of a specialized plasma membrane nucleoside transporter [4]. Gemcitabine is transported into the cell mainly by human equilibrative transporter 1 (hENT1). After intracellular entry, gemcitabine is phosphorylated by deoxycytidine kinase (dCK) to its active diphosphate and triphosphate in a rate-limiting step. The incorporation of gemcitabine triphosphate into DNA, leading to chain termination, is a major mechanism underlying the cytotoxicity of gemcitabine [5,6]. In addition, gemcitabine diphosphate inhibits ribonucleotide reductase (RRM1, RRM2), causing a decrease in the cellular pool of deoxycytidine triphosphate that competes with gemcitabine triphosphate for incorporation into DNA [7].

Recent investigations using cell lines or surgical specimens have revealed that these proteins were predictors for the efficacy of gemcitabine treatment. In particular, hENT1 expression has been evaluated as a predictive marker for gemcitabine chemotherapy in patients with pancreatic cancer. *In vitro* studies demonstrated that deficiency of hENT1 conferred resistance to gemcitabine and hENT1 expression was positively associated with gemcitabine chemosensitivity [4,8,9]. In patients with pancreatic cancer from a randomized phase III RTOG 9704 study, hENT1 expression was associated with increased overall survival (OS) and disease-free survival in patients who received adjuvant gemcitabine chemotherapy, but not in those who received 5-fluorouracil [10]. Other retrospective studies have also demonstrated the predictive and prognostic value of hENT1 in patients with pancreatic cancer [11,12].

Other key enzymes involved in gemcitabine metabolism have also been evaluated as predictive markers. In pancreatic cancer, high dCK expression was identified as an independent prognostic factor in patients who received adjuvant gemcitabine therapy [13]. In contrast, high expression of RRM1 was associated with poor survival after gemcitabine treatment in patients with recurrent pancreatic cancer [14,15].

There are thus many reports about predictive markers for the efficacy of gemcitabine in pancreatic cancer, but there are limited data available on the predictive value of these markers in BTC. Based on the importance of the biomarkers involved in gemcitabine metabolism, we assessed the expressions of three key molecules (hENT1, dCK, and RRM1) in tumor samples from 28 patients with advanced BTC who received first-line gemcitabine monotherapy. To our knowledge, this study is the first to examine the predictive aspect of hENT1, dCK, and RRM1 for gemcitabine-treated advanced BTC in the same clinical samples. The aim of this study was to investigate the association between the expressions of these proteins and prognosis.

## Methods

### Subjects

A total of 28 patients with histopathologically confirmed unresectable or postoperative recurrent BTC treated with first-line gemcitabine monotherapy at Osaka City University Hospital between October 2006 and April 2011 were included in this study. Adjuvant chemotherapy including gemcitabine was not given to these patients. BTC comprised extrahepatic bile duct cancer and gallbladder cancer. Intrahepatic bile duct cancer was excluded because unresectable or recurrent intrahepatic bile duct cancer patients in our institution were treated mostly with systemic gemcitabine plus hepatic arterial infusion of 5-fluorouracil. Subject demographics and clinical characteristics are listed in Table 1. In six subjects with unresectable cases, one had liver metastasis and five had peritoneal disseminations. The median OS from initiation of gemcitabine chemotherapy was 10.0 months for all 28 subjects. All tumor samples were obtained prior to gemcitabine chemotherapy. For all six unresectable tumors, the biopsy specimens were obtained from metastatic lesions during probe laparotomies. Chemotherapy consisted of intravenous gemcitabine infusion using the following protocols: the gemcitabine standard protocol (1,000 mg/m^2^ on days 1, 8, and 15 every 4 weeks). When the patients treated with weekly gemcitabine presented a grade 3 hematological adverse event or a grade 2 nonhematological adverse event defined by Common Terminology Criteria for Adverse Events version 3.0, a biweekly protocol (1,000 mg/m^2^ on days 1 and 15 every 4 weeks) was given to them. Informed consent to use the specimens for this study according to the institutional rules of the hospital was obtained from all subjects.

**Table 1:** Subject characteristics

**Total number**	**28**
Age (years)	
Median	64
Range	46 to 81
Gender	
Male	15
Female	13
Primary tumor site	
Extrahepatic bile duct	18
Gallbladder	10
Recurrence or unresectable	
Recurrence after surgery	22
Unresectable	6

### Immunohistochemistry of the specimens

Biomarker (hENT1, dCK, and RRM1) expression in BTC specimens was determined by immunohistochemical staining using the avidin–biotin–peroxidase complex method. In brief, the 4 μm thick formalin-fixed, paraffin-embedded sections were deparaffinized in xylene and decreasing concentrations of ethanol. The slides were treated with 3% hydrogen peroxide in methanol for 15 minutes to block endogenous peroxidase activity. To retrieve the antigenicities, slides were heated for 10 minutes at 105°C by autoclave in Target Retrieval Solution (Dako Co., Carpinteria, CA, USA) and cooled at room temperature for at least 30 minutes. Incubation was performed in a humidified chamber overnight at 4°C with anti-hENT1 rabbit polyclonal antibody (Proteintech, Chicago, IL, USA) at a 1:100 dilution and with anti-RRM1 mouse mAb (Proteintech) at a 1:100 dilution. The final incubation was for 2 hours at room temperature with anti-dCK rabbit polyclonal antibody (LSBio, Seattle, WA, USA) at a 1:24 dilution (20 μg/ml); 3,3′-diaminobenzidine (Nichirei, Tokyo, Japan) was used as a chromogen. The sections were rinsed, counterstained with hematoxylin, dehydrated through graded alcohol and xylene, and coverslipped. Negative controls processed by omitting the primary antibodies were included for each staining.

### Immunohistochemical evaluation

As previously reported [10], the scoring for hENT1 was based on relative intensities of the BTC staining with reference to the normally strong hENT1 staining within lymphocytes. These internal references were used as internal positive controls between slides and samples, as well as for the staining procedure. Tumor tissue staining was then evaluated by comparison with the internal controls. A score of high hENT1 staining was given for weak and/or strong reactivity in >50% of neoplastic cells. A score of low hENT1 staining was given if there was no staining in >50% of cells. Scoring for dCK and RRM1 was done in the same manner on the basis of the relative intensities of tumor staining with reference to the internal controls. The internal control for dCK was provided by lymphocyte staining as previously described [13]. Plasma and stromal cells showed positive RRM1 staining for cytoplasm, which was considered the internal control [16]. Staining grade was evaluated by two investigators without previous knowledge of the clinical characteristics and outcomes.

### Statistical analysis

All subjects were classified into high and low expression groups according to hENT1, dCK, and RRM1 staining. The significance of the correlation between expressions and clinicopathological characteristics was assessed by the chi-square test (Fisher’s exact test). Survival probabilities were calculated using the Kaplan–Meier method, and the log-rank test was used for univariate survival analysis. If OS was defined as the period from the date of first diagnosis of BTC, various factors such as operative curability and resectability might affect the survival time. The purpose of this study was focused on the impact of gemcitabine sensitivity-related gene products. OS was therefore measured from the date of first treatment with gemcitabine to the date of death or last follow-up evaluation. Progression-free survival (PFS) according to clinical judgment was measured from the date of first treatment with gemcitabine to the date of first progression or death without any progressive disease. Data on survivors were censored at the last follow-up. We also performed a multivariate analysis using Cox proportional hazards modeling to measure correlations between clinicopathological variables and OS. For all tests, two-sided *P* values <0.05 were defined as statistically significant. The SPSS software program (SPSS Japan, Tokyo, Japan) was used for the analysis.

## Results

### Immunostaining and subject background

**Figure 1:**
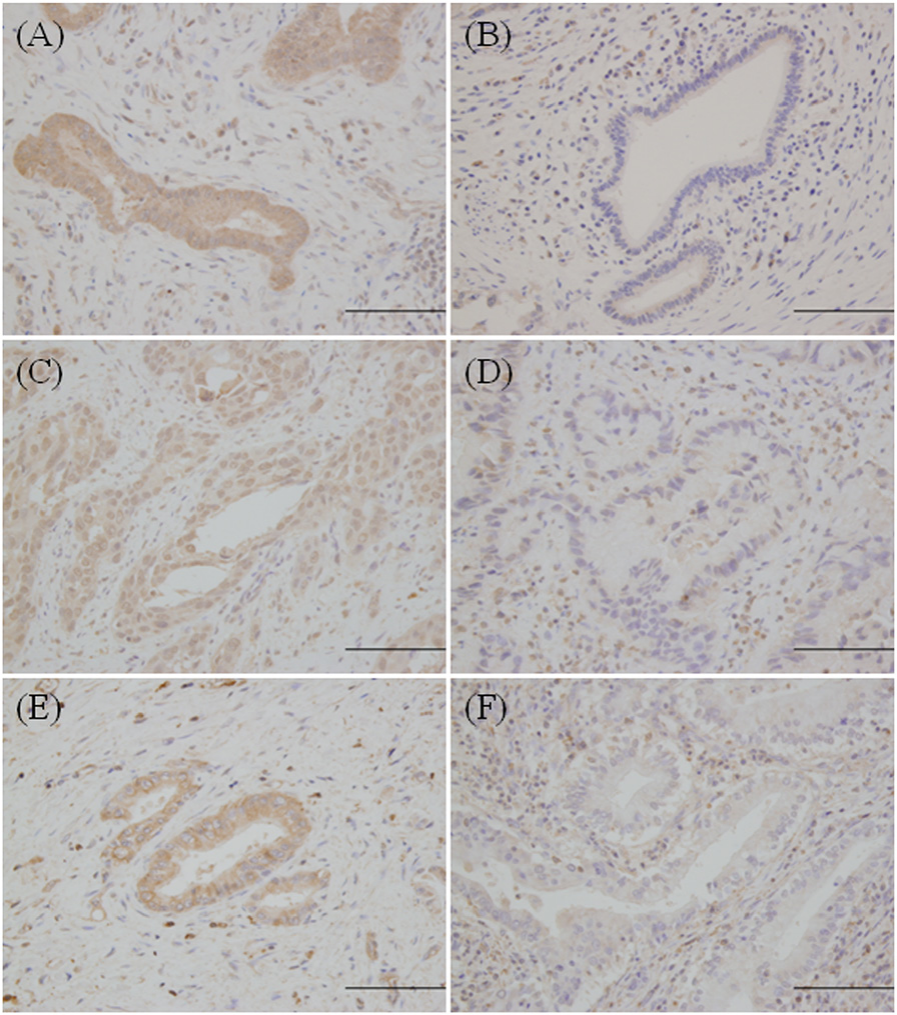
**Immunohistochemical analysis of hENT1, dCK, and RRM1 expressions in biliary tract cancer.** Representative immunohistochemical results. Human equilibrative nucleoside transporter 1 (hENT1): **(A)** high staining and **(B)** low staining. Deoxycytidine kinase (dCK): **(C)** high staining and **(D)** low staining. Ribonucleotide reductase subunit M1 (RRM1): **(E)** high staining and **(F)** low staining. Magnification × 200, scale bar = 100 μm.

**Figure 2:**
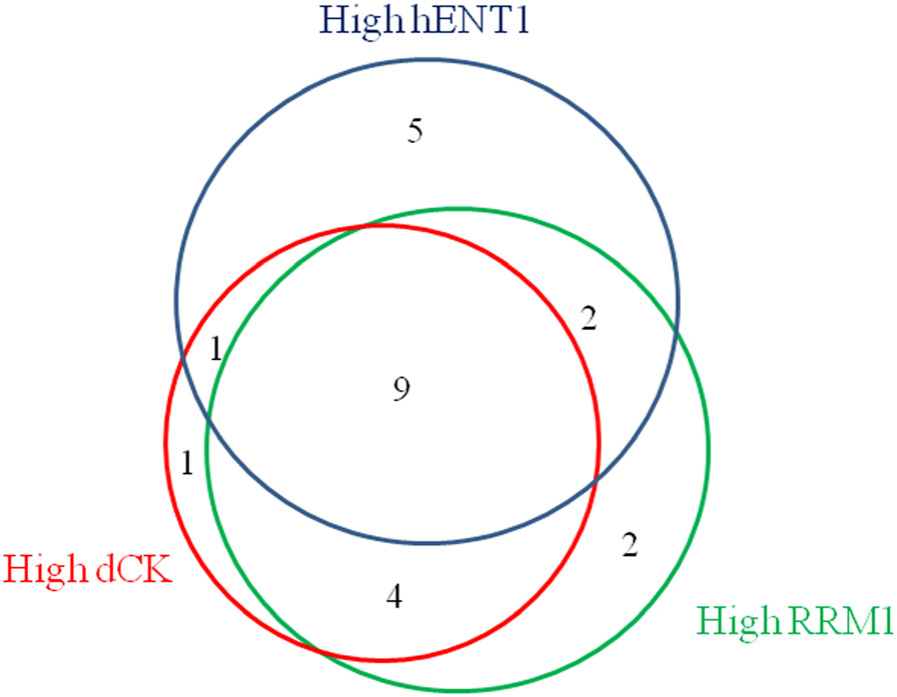
**Sample distribution for high hET1, dCK, and RRM1 expression in unresectable/recurrent biliary tract cancer.** The number in each area is the number of samples with high expression of these biomarkers. dCK, deoxycytidine kinase; hENT1, human equilibrative nucleoside transporter; RRM1, ribonucleotide reductase subunit M1.

Examples of high and low tumor staining are shown in Figure 1. hENT1 immunostaining was localized predominantly in the membrane, although occasional cytoplasmic staining was also observed. dCK immunostaining was located in the cytoplasm and the nucleus and RRM1 staining was seen in the cytoplasm. Of the 28 tumors, hENT1 was high in 17 (60.7%), dCK in 15 (53.6%), and RRM1 in 17 (60.7%). The relationship between clinicopathological factors and the expressions of hENT1, dCK, and RRM1 is summarized in Table 2. There were no significant differences in primary tumor site, recurrence/unresectable, or histological type. Among hENT1, dCK, and RRM1 expressions, a significant and strong association (*P* = 0.006) was observed between dCK and RRM1. Figure 2 shows the distribution of high expression of the three biomarkers.

**Table 2:** Clinicopathological characteristics based on hENT1, dCK, and RRM1 expression for advanced biliary tract cancer subjects

**Characteristic**	**hENT1 expression**		***P *value**	**dCK expression**		***P *value**	**RRM1 expression**		***P *value**
	**Low (*n *= 11)**	**High (*n *= 17)**		**Low (*n *= 13)**	**High (*n *= 15)**		**Low (*n *= 11)**	**High (*n *= 17)**	
Primary tumor site									
Extrahepatic bile duct	7	11	1.000	10	8	0.254	8	10	0.689
Gallbladder	4	6		3	7		3	7	
Recurrence or unresectable									
Recurrence after surgery	9	13	1.000	9	13	0.372	7	15	0.174
Unresectable	2	4		4	2		4	2	
Histological type									
Well/moderately	9	12	0.668	11	10	0.396	9	12	0.668
Poor	2	5		2	5		2	5	
hENT1 expression									
High	–	–	–	7	10	0.700	6	11	0.701
Low	–	–		6	5		5	6	
dCK expression									
High	*	*	*	–	–	–	2	13	0.006
Low	*	*		–	–		9	4	

dCK, deoxycytidine kinase; hENT1, human equilibrative nucleoside transporter; RRM1, ribonucleotide reductase subunit M1. *Same as above line (hENT1 expression).

### Survival analysis according to hENT1, dCK, and RRM1 expression

Univariate analysis showed that the expression of hENT1 was associated significantly with OS; however, primary tumor site, recurrence/unresectable, histological type, dCK, and RRM1 were not associated with OS (Table 3). Confounding factors (dCK and RRM1) could not be analyzed simultaneously in a multivariate analysis. Therefore, each of dCK and RRM1 was added to the multivariate analysis with primary tumor site, recurrence or unresectable, histological type, and hENT1 expression. The results demonstrated that high hENT1 expression alone was an independent prognostic predictor (Tables 4 and 5). Univariate analysis for PFS demonstrated significant association between hENT1 and PFS. Other variables including dCK and RRM1 were not related to PFS (Table 6).

**Table 3:** Univariate analysis for overall survival by log-rank test

**Variable**	**Number**	**Median overall survival (months)**	***P *value**
Primary tumor site			
Extrahepatic bile duct	18	11.4	0.2423
Gallbladder	10	9.2	
Recurrence or unresectable			
Recurrence	22	10.0	0.6183
Unresectable	6	9.2	
Histological type			
Well/moderately	21	10.0	0.9202
Poor	7	11.4	
hENT1 expression			
Low	11	5.7	0.0057
High	17	11.4	
dCK expression			
Low	13	9.2	0.3725
High	15	11.4	
RRM1 expression			
Low	11	10.2	0.5941
High	17	10.0	

dCK, deoxycytidine kinase; hENT1, human equilibrative nucleoside transporter; RRM1, ribonucleotide reductase subunit M1.

**Table 4:** Multivariate analysis for overall survival using variables including hENT1 and dCK

**Variable**	**Comparison**	**HR**	**95% CI**	***P *value**
Primary tumor site	Gallbladder vs. extrahepatic bile duct	1.617	0.596 to 4.389	0.345
Recurrence or unresectable	Unresectable vs. recurrence	1.707	0.511 to 5.700	0.384
Histological type	Poor vs. well/moderately	1.117	0.354 to 3.519	0.851
hENT1 expression	High vs. low	0.270	0.097 to 0.748	0.012
dCK expression	High vs. low	0.778	0.284 to 2.132	0.625

CI, confidence interval; dCK, deoxycytidine kinase; hENT1, human equilibrative nucleoside transporter; HR, hazard ratio.

**Table 5:** Multivariate analysis for overall survival using variables including hENT1 and RRM1

**Variable**	**Comparison**	**HR**	**95% CI**	***P *value**
Primary tumor site	Gallbladder vs. extrahepatic bile duct	2.136	0.751 to 6.078	0.155
Recurrence or unresectable	Unresectable vs. recurrence	3.109	0.860 to 11.238	0.084
Histological type	Poor vs. well/moderately	0.732	0.235 to 2.282	0.591
hENT1 expression	High vs. low	0.220	0.077 to 0.629	0.005
RRM1 expression	High vs. low	2.315	0.773 to 6.928	0.133

CI, confidence interval; hENT1, human equilibrative nucleoside transporter; HR, hazard ratio; RRM1, ribonucleotide reductase subunit M1.

**Table 6:** Univariate analysis for progression free survival by log-rank test

**Variable**	**Number**	**Progression-free survival (months)**	***P *value**
Primary tumor site			
Extrahepatic bile duct	18	4.6	0.2551
Gallbladder	10	3.4	
Recurrence or unresectable			
Recurrence	22	3.9	0.7073
Unresectable	6	7.4	
Histological type			
Well/moderately	21	4.4	0.7255
Poor	7	5.2	
hENT1 expression			
Low	11	2.5	0.0065
High	17	7.7	
dCK expression			
Low	13	4.6	0.8076
High	15	3.7	
RRM1 expression			
Low	11	7.4	0.1349
High	17	2.5	

dCK, deoxycytidine kinase; hENT1, human equilibrative nucleoside transporter; RRM1, ribonucleotide reductase subunit M1.

## Discussion

hENT1 is the primary gatekeeper for intracellular uptake of gemcitabine, and both dCK and RRM1 are related to gemcitabine metabolism after intracellular entry. Marechal and colleagues recently investigated the predictive value of hENT1, dCK, and RRM1 in patients with pancreatic cancer treated with adjuvant gemcitabine chemotherapy and demonstrated that both hENT1 and dCK expressions were powerful predictive markers [17]. There have been several studies on the impact of single gemcitabine sensitivity-related gene products (such as hENT1, dCK, and RRM1) on gemcitabine-based treatment effects in BTC. These previous investigations were based on immunohistochemistry. To our knowledge, there have been no studies on the association between gemcitabine effects and mRNA expression of gemcitabine sensitivity-related genes in BTC. Our study was performed using the immunohistochemical method because the frozen tissues of BTC studied here were not kept. The originality of our study is that we observed all of these biomarkers simultaneously in patients with BTC treated only with gemcitabine.

We analyzed hENT1, dCK, and RRM1 expression using immunohistochemical analysis to determine the prognostic value in patients with advanced BTC treated with gemcitabine. hENT1 was the only independent predictive marker for gemcitabine chemotherapy in advanced BTC. Patients with a high level of tumor hENT1 had a statistically significant longer OS and PFS than those with low expression. Our study also demonstrated that high hENT1 expression was an independent prognostic factor in patients with advanced BTC, treated with first-line gemcitabine monotherapy.

There are a few papers on the prognostic value of hENT1 in patients with BTC treated with gemcitabine. Santini and colleagues reported that an immunohistochemical evaluation of intratumoral hENT1 expression might be useful in predicting the clinical outcome of gemcitabine-based chemotherapies in 31 patients with advanced BTC (extrahepatic biliary tract, intrahepatic biliary tract, gall bladder, and ampulla). However, they were unable to find a statistically significant difference in OS [18]. Borbath and colleagues reported that high expression of hENT1 was independently associated with prolonged PFS and OS in 26 patients with locally advanced or metastatic extrahepatic and intrahepatic cholangiocarcinoma who were treated with gemcitabine [19]. Kobayashi and colleagues recently demonstrated that high hENT1 expression was associated with prolonged OS in resected extrahepatic and intrahepatic cholangiocarcinoma treated with gemcitabine-based adjuvant chemotherapy [20]. The primary sites of BTC in these studies and ours varied. However, the positive survival impact of hENT1 on BTC treated with gemcitabine-based chemotherapy in these studies was concordant. On the other hand, the potential prognostic value of hENT1 expression in patients with BTC who did not receive gemcitabine chemotherapy is unclear. Although we investigated the expression of hENT1 in 39 patients with advanced BTC who did not receive gemcitabine, no significant survival difference was observed between patients with high and low hENT1 (data not shown).

RRM1 have been reported previously as predictive markers for gemcitabine in pancreatic cancer, although there are few published studies evaluating their value in BTC. Nakamura and colleagues reported that high RRM1 expression had a positive association with poor prognosis in 10 patients with advanced BTC treated with gemcitabine-based chemotherapy with or without radiation [21]. In the current study, the expression of RRM1 was not associated with a prognosis. The differing results for RRM1 impact on the survival of patients with BTC between Nakamura and colleagues’ study and ours might be due to the different treatment modalities.

An abundance of dCK is commonly known to be associated with gemcitabine sensitivity in pancreatic cancer. There has been no paper describing dCK expression in patients with BTC. In the current study, there was significant correlation between dCK and RRM1 expression. The reason for this is unclear, but both proteins are key enzymes related to deoxycytidine metabolism.

The limitation of this study is that it was a retrospective evaluation. Prospective investigation including an adequate number of samples is needed to confirm the importance of gemcitabine sensitivity-related gene products in patients with advanced BTC treated with gemcitabine. In addition, the relations between gemcitabine effects in BTC and other gemcitabine sensitivity-related gene products such as RRM2, cytidine deaminase, human concentrative nucleoside transporter 1 and 3, 5′-nucleotidase, and deoxycytidylate deaminase are remained to be elucidated for further investigations.

## Conclusions

In this study, high hENT1 expression was a reliable predictive marker of survival in patients with recurrent or unresectable BTC who received gemcitabine chemotherapy alone.

## Abbreviations

BTC: Biliary tract cancer; dCK: Deoxycytidine kinase; hENT1: Human equilibrative nucleoside transporter 1; mAb: Monoclonal antibody; OS: Overall survival; PFS: Progression-free survival; RRM1: Ribonucleotide reductase subunit M1.

## Competing interests

The authors declare that they have no competing interests.

## Authors’ contributions

AM carried out almost studies and performed the manuscript. RA and NY supported with design and interpretation of this study. Statistical analysis was performed by AM and KK. MY and BN helped to draft the manuscript. Overall supervision of the manuscript was completed by KH. All authors read and approved the final manuscript.
